# Mitochondrial and Peroxisomal Alterations Contribute to Energy Dysmetabolism in Riboflavin Transporter Deficiency

**DOI:** 10.1155/2020/6821247

**Published:** 2020-08-12

**Authors:** Fiorella Colasuonno, Alessia Niceforo, Chiara Marioli, Anna Fracassi, Fabrizia Stregapede, Keith Massey, Marco Tartaglia, Enrico Bertini, Claudia Compagnucci, Sandra Moreno

**Affiliations:** ^1^Department of Science, LIME, University of Roma Tre, Rome 00146, Italy; ^2^Department of Neuroscience, Unit of Neuromuscular and Neurodegenerative Diseases, Laboratory of Molecular Medicine, IRCCS Ospedale Pediatrico Bambino Gesù, Rome 00146, Italy; ^3^Genetics and Rare Diseases Research Division, IRCCS Ospedale Pediatrico Bambino Gesù, Rome 00146, Italy; ^4^Mitchell Center for Neurodegenerative Diseases, Department of Neurology, University of Texas Medical Branch, Galveston, 77550 TX, USA; ^5^Cure RTD Foundation, Calgary, Alberta, Canada

## Abstract

Riboflavin transporter deficiency (RTD) is a childhood-onset neurodegenerative disorder characterized by progressive pontobulbar palsy, sensory and motor neuron degeneration, sensorineural hearing loss, and optic atrophy. As riboflavin (RF) is the precursor of FAD and FMN, we hypothesize that both mitochondrial and peroxisomal energy metabolism pathways involving flavoproteins could be directly affected in RTD, thus impacting cellular redox status. In the present work, we used induced pluripotent stem cells (iPSCs) from RTD patients to investigate morphofunctional features, focusing on mitochondrial and peroxisomal compartments. Using this model, we document the following RTD-associated alterations: (i) abnormal colony-forming ability and loss of cell-cell contacts, revealed by light, electron, and confocal microscopy, using tight junction marker ZO-1; (ii) mitochondrial ultrastructural abnormalities, involving shape, number, and intracellular distribution of the organelles, as assessed by focused ion beam/scanning electron microscopy (FIB/SEM); (iii) redox imbalance, with high levels of superoxide anion, as assessed by MitoSOX assay accompanied by abnormal mitochondrial polarization state, evaluated by JC-1 staining; (iv) altered immunofluorescence expression of antioxidant systems, namely, glutathione, superoxide dismutase 1 and 2, and catalase, as assessed by quantitatively evaluated confocal microscopy; and (v) peroxisomal downregulation, as demonstrated by levels and distribution of fatty acyl *β*-oxidation enzymes. RF supplementation results in amelioration of cell phenotype and rescue of redox status, which was associated to improved ultrastructural features of mitochondria, thus strongly supporting patient treatment with RF, to restore mitochondrial- and peroxisomal-related aspects of energy dysmetabolism and oxidative stress in RTD syndrome.

## 1. Introduction

Flavins are a family of ubiquitous water-soluble compounds, sharing the basic structure of 7,8-dimethyl-10-alkylisoalloxazine and participating in many biochemical reactions as coenzymes [1]. Riboflavin (RF), also known as vitamin B2, is the precursor of all biologically important flavins, and it is widely distributed throughout the plant and animal kingdoms [2]. Flavins are broadly distributed in tissues, but the majority is found in flavocoenzymes, mainly as flavin adenine dinucleotide (FAD) and in lesser amounts as flavin mononucleotide (FMN).

Ever since their discovery and characterization, RF and its derivatives have been recognized by their ability to transfer single electrons, hydrogen atoms, and hydride ions, thus making flavoenzymes highly versatile, in terms of substrate modifications and types of reactions. The direct involvement of FAD and FMN, as rate limiting factors in energy metabolism, is widely accepted, as well as the direct correlation between the rate of cellular energy metabolism and the levels of FAD and FMN [3–5].

RF is essential for a wide variety of reactions, which preferentially occur in mitochondria [6, 7]. The oxidative metabolism of carbohydrates, fatty acids, amino acids, choline, and several other bioenergetically relevant metabolites depends on flavoproteins functionality. Specifically, subunits of the respiratory chain complexes I and II, as well as the electron transfer flavoprotein and its ubiquinone oxidoreductase, localized to the inner mitochondrial membrane, drive electrons from several reduced flavoproteins to ubiquinone and then to complex III of the respiratory chain [8].

Several metabolic pathways depending on flavoprotein functionality occur in peroxisomes, which contain more than 50 flavoenzymes functioning in crucial steps of anabolic and catabolic reactions [9]. Peroxisomes play key cooperative roles in the metabolism of cellular lipids and reactive oxygen species (ROS), making them indispensable for human development and health [10]. Mammalian peroxisomes contribute to the breakdown and detoxification of fatty acids (via fatty acid *α* and *β*-oxidation); the synthesis of ether-phospholipids (e.g., myelin sheath lipids), bile acids, and docosahexaenoic acid; glyoxylate metabolism; amino acid catabolism; polyamine oxidation; and reactive nitrogen species (RNS) metabolism [11]. Over the last decades, peroxisome crosstalk with mitochondria, the endoplasmic reticulum, lipid droplets, lysosomes, and phagosomes has received growing attention [12]. Recent evidence also indicates functional interplay between peroxisomes and the nucleus, which may also involve signaling via H_2_O_2_ [13].

Mammals, including humans, have lost the ability to synthesize water-soluble vitamins, and they obtain these compounds from exogenous sources to prevent clinical signs of deficiency [14], the minimal requirement for RF being around 0.35 mg/1,000 kcal. Absorption in mammals takes place mainly in the small intestine and partly in the large intestine [8]. The physiological mechanisms regulating RF uptake by the different mammalian organs and tissues are only partially understood, but it has recently been demonstrated that RF transport is carrier mediated [8, 15, 16].

Three human riboflavin transporters belonging to the SLC52 family of solute carriers have been characterized. Recent nomenclature refers to them as hRFVT1, hRFVT2, and hRFVT3, encoded by the *SLC52A1*, *SLC52A2*, and *SLC52A3* genes [15, 16]. hRFVT1and hRFVT2 proteins share 86% amino acid identity, while they only exhibit 42% and 43% identity with hRFVT3, respectively. The three proteins have partially overlapping subcellular and tissue-specific expression profiles. Both hRFVT2 and hRFVT3 are widely distributed, with hRFVT3 having the highest expression in the testis, small intestine, kidney, and placenta and hRFVT2 having the highest expression in the brain and spinal cord [17–19]. In contrast, hRVFT1 has little expression in the central nervous system with the highest expression in the small intestine and placenta [20]. They are predicted to have 10 (hRFVT1 and 2) or 11 (hRFVT3) membrane-spanning domains [21].

A clinically wide spectrum of human diseases has been associated with defective function of flavoproteins, including Leigh syndrome and other mitochondrial respiratory chain disorders, mitochondrial and peroxisomal fatty acid oxidation (FAO) disorders, pyridoxal phosphate responsive epilepsy, trimethylaminuria, rhizomelic chondrodysplasia punctata, porphyria variegata, chronic granulomatous disease, and defects of steroidogenesis and bile acid synthesis [5].

RF deficiency during pregnancy and adolescence may be related to developmental abnormalities and to an increased risk for anemia, cancer, and cardiovascular and neurodegenerative diseases [22]. Moreover, human metabolic diseases affecting nervous and muscular systems are caused by flavoprotein derangements or inadequate availability of flavin cofactors caused by genetic mutations [3, 23]. Among these, there are the RF-responsive multiple Acyl-CoA dehydrogenase deficiency, the Fazio-Londe syndrome, and the riboflavin transporter deficiency (RTD).

RTD syndrome, formerly known as Brown-Vialetto-Van Laere (BVVL) syndrome, is a rare autosomal-recessive motor neuron disease. Typical clinical features reported include pontobulbar palsy, limb and axial muscle weakness, sensorineural hearing loss, optic atrophy, ataxia, and respiratory compromise [24, 25]. The age of onset is variable, and young patients show different phenotypes with variable prognosis, often influenced by the initiation of RF treatment. In fact, clinical evidence brought by Bosch and coll. [23] argues for partial amelioration of the pathological phenotype of RTD patients, after high-dose vitamin B2 administration.

Numerous mutations in *SLC52A2* and *SLC52A3* have been recognized as causative for the childhood-onset RTD syndrome [26]. Lack of association of a pathological condition with mutations in *SLC52A1* likely indicates an indispensable role of this transporter early in development [16, 27].

When *SLC52A2* and *SLC52A3* RF transport into the cell is reduced, its limited availability can compromise several vital processes [28, 29]. It is therefore likely that mitochondria and peroxisomes are impaired in their integrity and bioenergetics in RTD syndrome. Unfortunately, *in vivo* models for RTD, recapitulating symptoms and progression, are so far lacking. RF-deficient mouse models have been generated but are characterized by embryonic or early postnatal lethality, which does not allow studies on the pathological phenotype [30, 31].

In the present work, we took advantage of the induced pluripotent stem cell (iPSC) technology to shed light on the molecular mechanisms and dysfunctional cellular processes underlying this severe neurological disorder. Such technology is well consolidated in biomedical research, since it allows the development of patient-specific cellular models, generally preserving genome integrity [32], even in the presence of epigenetic remodeling [33]. On the other hand, morphofunctional changes occurring in cytoplasmic organelles, particularly mitochondria, during cell reprogramming and maintenance, are now widely characterized, allowing the researchers to make comparisons between healthy and diseased conditions [34–36].

Human iPSCs from RTD patients carrying *SLC52A2* gene mutations were characterized, focusing on mitochondrial and peroxisomal alterations. The characterized morphological, ultrastructural, and molecular endophenotype was used to assess the effects of RF supplementation as treatment to rescue such dysfunctions. These experiments were aimed at providing the mechanistic basis at the cellular level for the efficacy of symptomatic, empirical therapies proposed for RTD [23, 37–43].

## 2. Materials and Methods

### 2.1. Derivation of iPSCs and Treatment

Human iPSCs were derived from fibroblasts of healthy subjects and two RTD patients carrying *SLC52A2* gene mutations (c.155C>T and c.935T>C; c.155C>T and c.1255G>A). One patient (P1) presented at 3 months of age with macrocytic anemia and dysphagia. Optic atrophy, axial muscle weakness, sensory ataxia, and respiratory compromise were noted at 1 year and bilateral sensorineural hearing loss at 2 years. Patient P1 is 9 years old and appears neurologically stable since beginning riboflavin (75 mg/kg QID) and antioxidant therapy at 2.5 years of age. The second patient (P2), as reported by Ciccolella and coll. [44], developed progressive dysphonia and notable exercise intolerance with dyspnea and cyanosis at 2 years, bilateral sensorineural hearing loss, reduced visual acuity, and progressive shoulder and axial muscle weakness at 3 years. Before the 4^th^ year, P2 required hospitalization for acute respiratory failure and aspiration pneumonia and after a few days, the patient died [44].

Fibroblasts were reprogrammed by using the nonintegrating episomal technology (Epi Episomal iPSC Reprogramming Kit, Cat. no. A15960, ThermoFisher Scientific, Waltham, MA, USA) by System Biosciences (Palo Alto, CA, USA). Cells were grown under feeder-free conditions, using Matrigel (BD Biosciences, NJ, USA) in mTeSR1 (Stem Cell Technologies Inc.), and incubated at 37°C, in hypoxic conditions.

Control (Ctrl) and patients' iPSCs were treated with the culture medium supplemented or not with 1 *μ*M RF (R9504, Sigma-Aldrich, St. Louis, MO, USA) overnight.

### 2.2. DNA Sequencing

Ctrl and RTD iPSC pellets, obtained by centrifuging EDTA-detached cells at 1200 rpm for 5 min, were processed for genomic DNA extraction (Qiagen, Hilden, Germany) following the manufacturer's instructions.

The coding sequence of exons 3 and 5 of the *SLC52A2* gene (NM_001363118) was amplified by PCR specific primers. Amplimers were purified using Exo-SAP (GE Healthcare) and directly sequenced using BigDye 3.1 chemistry (Applied Biosystems, Foster City, CA, USA) with an ABI Prism 3130 xl automatic sequencer (Applied Biosystems).

### 2.3. Light and Confocal Microscopy

General morphological features of cell cultures were assessed by an inverted microscope Olympus IX Leica (Tokyo, Japan), equipped with IAS 2000 image capturing software.

For confocal analysis, cells were fixed with 4% paraformaldehyde for 10 min at room temperature (RT), washed with PBS, and blocked with 5% bovine serum albumin and 0.1% Triton X-100 (Sigma-Aldrich). Polyclonal or monoclonal primary antibodies against the following are used: peroxisomal markers (1 : 200 catalase (anti-CAT, 100-4151, Rockland Immunochemicals, Inc., Limerick, PA, USA), 1 : 250 acyl-CoA oxidase (anti-ACOX, ab184032, Abcam, Cambridge, UK), 1 : 200 PMP70 (P0497, Sigma-Aldrich), and 1 : 200 ACAA1 (ab154091, Abcam)); mitochondrial marker (1 : 200 Mitochondria Antibody (MTC02) (NB600-556, Novus Biological, Centennial, CO, Stati Uniti); antioxidant molecules (1 : 200 superoxide dismutase 1 (anti-SOD1, ab13498, Abcam), 1 : 200 superoxide dismutase 2 (anti-SOD2, ab13533, Abcam), 1 : 100 glutathione (anti-GSH, 101-A, Virogen, Watertown, MA, USA), and 1 : 100 zonula occludens-1 (anti-ZO-1 (33-9100, ThermoFisher Scientific)).

After washings in PBS, cells were incubated for 1 h with the appropriate secondary antibodies, conjugated with Alexa Fluor 488 or Alexa Fluor 594 (Invitrogen, Carlsbad, CA, USA), and diluted 1 : 500 in PBS. Nuclei were stained with 1 *μ*g/ml Hoechst (33342, Invitrogen) for 10 min at RT, and coverslips were mounted using 1 : 1 PBS/glycerol. Slides were observed in a Leica TCS SP5 confocal microscope (Leica, Wetzlar, Germany). Representative images, captured by a Leica Application Suite software, were composed in an Adobe Photoshop CS6 format (Adobe Systems Inc., San Jose, CA, USA).

### 2.4. Ultrastructural Analyses

For focused ion beam/scanning electron microscopic (FIB/SEM) analyses, cells were plated and treated in a chamber slide (Lab-Tek™ II Chamber Slide System, ThermoFisher Scientific) and fixed in 0.5% glutaraldehyde and 2% paraformaldehyde, in 0.1 M cacodylate buffer, pH 7.4, for 1 h, at 4°C. Postfixation with osmium tetroxide, dehydration, and embedding in epoxy resin were performed as previously described [45]. Resin-embedded cells were mounted on stubs using a self-adhesive carbon disk and gold sputtered by an Emitech K550. Samples were then analyzed by a DualBeam FIB/SEM (Helios Nanolab, FEI, Hillsboro, OR, USA). Regions of interest were cross sectioned by the focused gallium ion beam of the device. Micrographs were acquired at a working distance of 2 mm using backscattered electrons and a through-the-lens detector in immersion mode with an operating voltage of 2 kV and an applied current of 0.17 nA.

For SEM analysis, cells were plated on glass slides; fixed in 2.5% glutaraldehyde, for 1 h; postfixed in 1% osmium tetroxide, for 45 min; and gradually dehydrated (from 50% to 100% ethanol). Air drying was performed using hexamethyldisilazane and, once mounted on stubs, a sputter coater was used to make the sample properly conductive. Micrographs were acquired at a working distance of 2 mm using backscattered electrons and both Everhart-Thornley and through-the-lens detectors (ETD and TLD) with an operating voltage of 2 kV and an applied current of 0.34 nA. Images were assembled in Adobe Photoshop CS6 software (Adobe Systems Inc.).

### 2.5. MitoSOX Red Assay

Detection of O_2_^−^ was performed by MitoSOX Red Mitochondrial Superoxide Indicator (Molecular Probes, Eugene, OR, USA). This fluorogenic dye for highly selective detection of O_2_^−^ in the mitochondria measures the fluorescence intensity increase (emission: ~510/580 nm). Cells were preliminarily detached with EDTA, centrifuged at 1200 rpm for 5 min, washed in PBS, and solubilized in Mitochondria Incubation Buffer (Molecular Probes). Cells were incubated with 5 *μ*M MitoSOX Red Probe for 45 min at 37°C. The fluorescence intensity was measured by an EnSpire Multimode Plate Reader (Perkin Elmer, Boston, MA, USA). Data were normalized based on protein amount determined by the bicinchoninic acid assay (Pierce BCA Protein Assay Kit, ThermoFisher Scientific).

### 2.6. JC-1 Staining

5,5,6,6′-Tetrachloro-1,1′,3,3′-tetraethylbenzimidazolylcarbocyanine chloride (JC-1) (T3168, Life Technologies, Carlsbad, CA, USA) probe was used to monitor mitochondrial membrane potential (MMP), according to the manufacturer's instructions. Cells were grown in mTeSR1 and treated with 5 *μ*M JC-1 for 30 min at 37°C. After washing twice with mTeSR1, cells were photographed before and after 1, 5, 10, 15, and 30 min following 10 *μ*M H_2_O_2_ exposure. Regions of high mitochondrial polarization are revealed by red fluorescence due to J-aggregate formation of the concentrated dye, whereas depolarized regions are indicated by green fluorescence of JC-1 monomers. Pictures are representative images of four experiments performed in live imaging condition using an inverted Leica DMi8 microscope (Leica,) where CO_2_ (5%) and temperature (37°C) were controlled using a Top Stage Incubator (Okolab Srl, Italy). Time course of iPSCs before and during H_2_O_2_ treatment shows the fluorescence intensity ratio for JC-1 staining (red/green fluorescence intensity ratio).

### 2.7. Statistical Analysis

For mitochondrial morphometric analyses, 3 samples/cell culture type and 10 cells/sample were analyzed by manually counting regular and altered mitochondria of Ctrl and RTD iPSCs and calculating their respective percentage. The mean diameter of mitochondria was obtained by manually measuring the organelles in the same samples.

Statistical analysis was performed using Prism software (GraphPad Software, Inc., La Jolla, CA, USA). The results are presented as means ± SD of *n* ≥ 3 independent experiments.

For MitoSOX Red assay, Mann-Whitney test was used. For all the other data, one-way ANOVA was used to analyze differences among genetic conditions and treatments, followed by Tukey's multiple comparison test or Bonferroni test, as appropriate. A *p* value of 0.05 or less was considered statistically significant (^∗^), while ∗∗ and ∗∗∗ indicate *p* values equal or lower than 0.01 and to 0.001, respectively.

Diagrams were assembled using PowerPoint and Photoshop CS6.

## 3. Results and Discussion

### 3.1. Results

**Figure 1:**
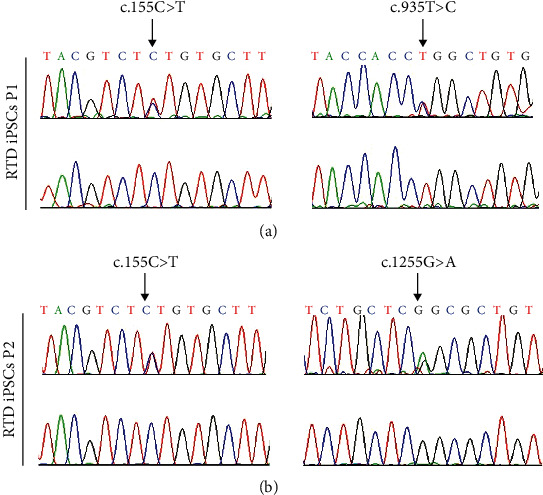
Sanger sequencing in Ctrl and RTD iPSCs. Electropherograms show the pathogenic *SLC52A2* mutations identified in RTD iPSCs of P1 ((a), upper) and RTD iPSCs of P2 ((b), upper). The arrows indicate the position of the mutation. Ctrl iPSC ((a, b), lower) result is negative for each mutation.

The morphofunctional features of iPSCs from two RTD patients, carrying composite heterozygous mutations (c.155C>T and c.935T>C; c.155C>T and c.1255G>A) in the *SLC52A2* gene were examined. The identified mutations were confirmed by Sanger sequencing on DNA extracted from RTD iPSC pellets, while Ctrl is negative for the mutations (Figure 1).

Morphological studies were performed by a panel of light and electron microscopic techniques. These were followed by functional analyses, with molecular probes, focusing on mitochondrial and peroxisomal compartments.

#### 3.1.1. Phase-Contrast, Confocal, and Electron Microscopic Analyses of iPSCs

**Figure 2:**
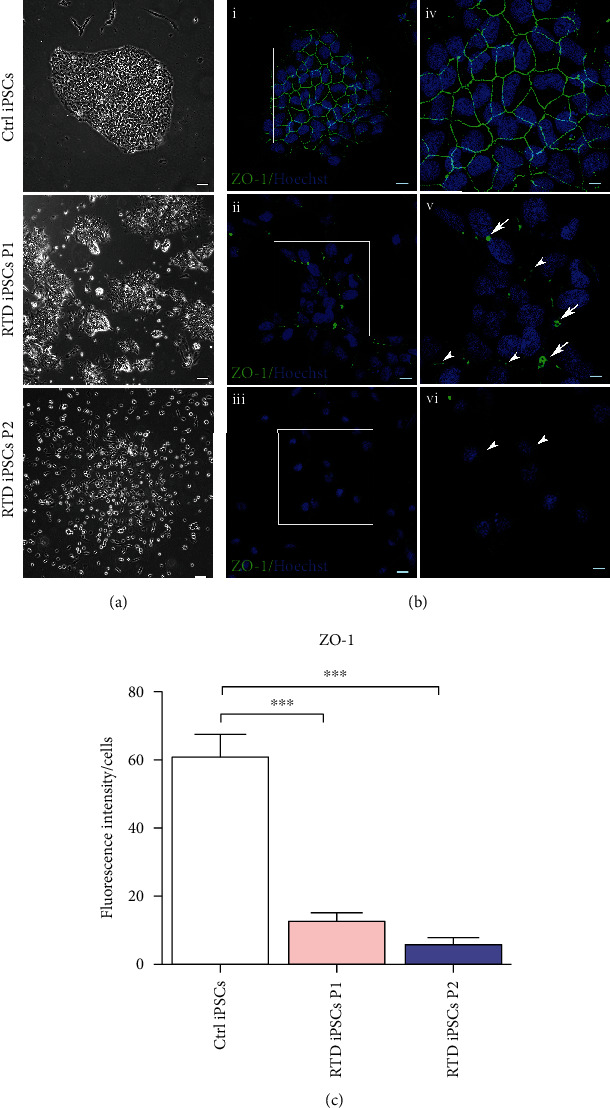
Morphological features of Ctrl and RTD iPSCs. (a) Phase contrast images show absence of typical colonies in diseased cells as compared to controls. Scale bars, 20 *μ*m. (b) Confocal analysis after immunofluorescence using ZO-1 marker (green) demonstrates abnormal cell-cell contacts in RTD (ii, iii, v, and vi) as compared to Ctrl (i, iv) iPSC. White arrows indicate ZO-1 aggregates in RTD iPSCs P1 (vi, vii) while white arrowheads indicate immunofluorescence puncta (v, vi). Scale bars, 10 *μ*m (i–iii), 5 *μ*m (iv–vi). Nuclei are stained with Hoechst (blue). (c) Fluorescence intensity levels reveal a significant decrease of ZO-1 expression in RTD vs. Ctrl iPSCs (^∗∗∗^*p* ≤ 0.001).

When compared to Ctrl cells, patient-derived iPSC cultures exhibit morphological defects, including absence of typical, roundly shaped colonies, and large extracellular spaces (Figure 2(a)). Spatial relationships among cells were further inspected by confocal immunofluorescence analysis using anti-ZO-1 (Figure 2(b)). While regular distribution of this tight junction protein at plasma membranes level is detected in Ctrl cells, unusual localization in few, large puncta or aggregates is observed in P1 cells (arrows in (ii)), and almost lack of staining is found in P2-derived cells. These abnormalities are even more apparent in higher magnification micrographs, showing details of ZO-1 distribution (Figure 2(b) (iv–vi)). Accordingly, statistical analysis demonstrates significantly lower ZO-1 fluorescence intensity in cell cultures from both patients, with respect to Ctrl cells (Figure 2(c)).

**Figure 3:**
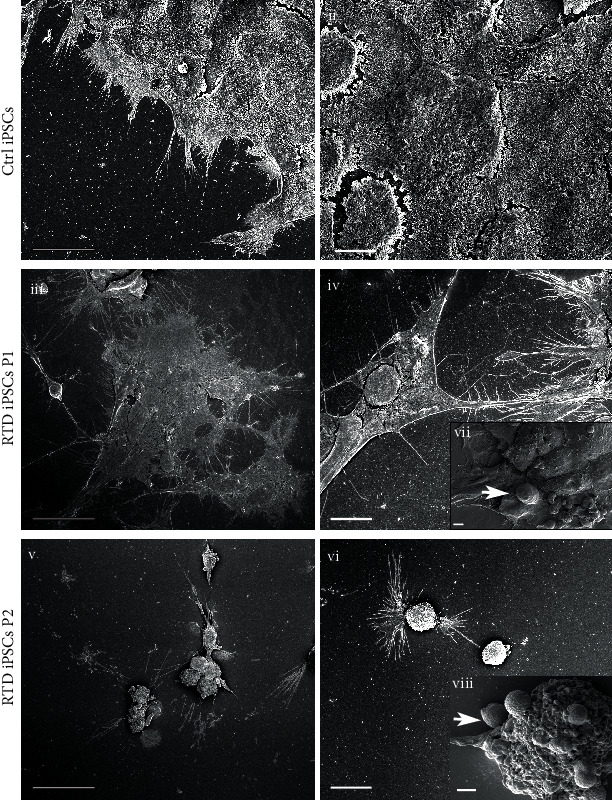
SEM micrographs showing different morphology of RTD iPSCs, as compared to controls. Ctrl iPSCs (i, ii) organize in well-defined colonies, while RTD iPSCs (iii–vi) are found in small colonies or as single cells, often extending lamellipodia (only in P1) or filopodia (in both P1 and P2). P1 cells generally appear more flattened in shape (iii), while P2 cells show a more roundish shape (v), with several vesicles (~1 *μ*m in diameter) budding from the membrane (viii). Accumulation of extracellular vesicles in aciniform aggregates at the apical cell surface (white arrows) is also observed in higher magnification images captured using a TDL detector (vii, viii) (see Material and Methods for details). Scale bars, 25 *μ*m in (i), (iii), and (v); 10 *μ*m in (ii), (iv), and (vi); 1 *μ*m in (vii) and (viii).

Scanning electron microscopy (SEM) was also used to analyze details of RTD iPSC surface (Figure 3). Unlike Ctrl iPSCs, patient-derived iPSC cultures organize in small colonies (mostly in P1) or as single cells (mostly in P2), often extending lamellipodia (only in P1) or filopodia (in both P1 and P2), to form focal adhesions with the substrate. P1 cells generally appear more flattened in shape, while P2 cells show a more roundish shape with several vesicles (~1 *μ*m in diameter) budding from the membrane. Accumulation of extracellular vesicles in aciniform aggregates at the apical cell surface is also observed, particularly in P2 (Figure 3 (vii–viii)).

**Figure 4:**
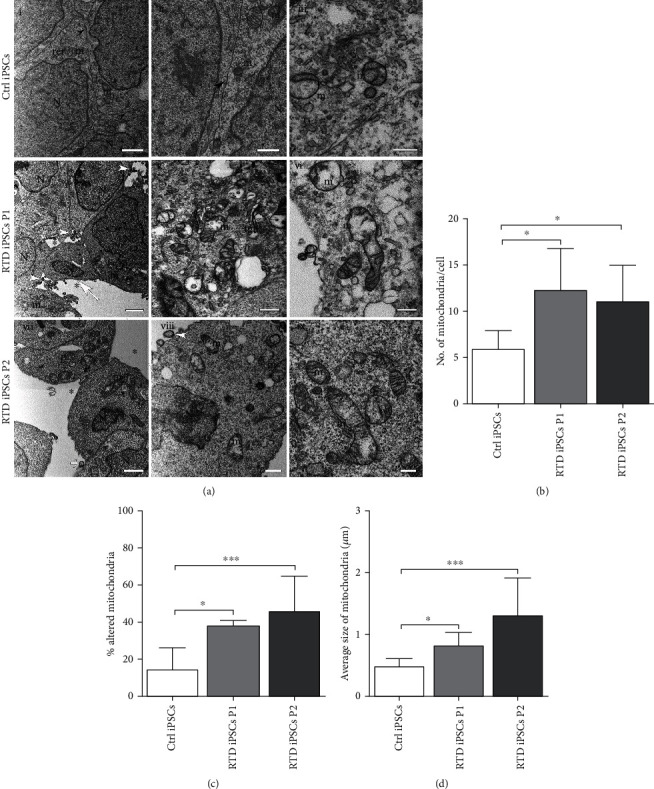
Ultrastructural features of Ctrl and RTD iPSCs. (a) FIB/SEM micrographs showing abnormal cell-cell contacts in RTD iPSC cultures (iv, vii), as compared to Ctrl cells (i, ii) which display recognizable tight junctions (black arrowheads in (i) and (ii)). RTD cell cultures display loose cell-cell contacts and large intercellular spaces (asterisks in (iv) and (vii)). White arrowheads indicate extracellular vesicles, in proximity to plasma membrane protrusions in RTD cells (iv–vii); white arrow in (iv) indicates a membrane-limited cell fragment containing nuclear parts. Ctrl iPSCs show few healthy immature mitochondria with poorly developed *cristae* (ii, iii) while several damaged organelles with disrupted cristae are observed in RTD iPSCs (P1 and P2) (v, vi, viii, and ix). N: nuclei; m: mitochondria; rer: rough endoplasmic reticulum. Scale bars, 2 *μ*m in (i), (iv), and (vii); 1 *μ*m in (ii), (v), and (viii); 0.5 *μ*m in (iii), (vi), and (ix). (b) Mitochondria are significantly more numerous in patients' iPSCs than in Ctrl. (c) Patients' iPSCs show a significantly higher number of damaged mitochondria, compared to Ctrl. (d) Mitochondrial average size is significantly larger in patients' iPSCs than in Ctrl.

Consistent with the above morphological results, focused ion beam/scanning electron microscopy (FIB/SEM) reveals abnormal cell-cell contacts in RTD iPSC cultures, as compared to Ctrl (Figure 4(a)). Different from the long stretches of juxtaposed plasma membranes, with recognizable tight junctions (arrowheads in (i) and (ii)) shown by healthy cells, loose cell-cell contacts with few adherent junctions are displayed by P1 and P2 cells, with large intercellular spaces characterizing both cell lines (asterisks in (iv) and (vii)). Extracellular vesicles, in proximity to plasma membrane protrusions, are often observed (higher magnification micrographs in (vii) and (viii)). Moreover, membrane-limited cell fragments containing nuclear parts (white arrow in (ii)) are found in RTD cultures.

Ultrastructural analysis also highlights marked mitochondrial abnormalities associated to the RTD phenotype, involving shape, number, and intracellular distribution. These organelles appear elongated, sometimes swollen, with disrupted or even absent *cristae* (Figure 4(a) (iv–ix)). All these features are lacking in Ctrl cells, which instead display roundly shaped, small, immature mitochondria with poor-developed *cristae* (Figure 4(a) (i–iii)). Notably, mitochondria of patients' iPSCs appear more dispersed in the cytoplasm, as compared to those of Ctrl cells, which are mainly localized near the nucleus. Statistical analysis to evaluate the occurrence of normal and altered mitochondria and their average size was also performed (Figures 4(a)–4(d)). Data demonstrate the presence of significantly more numerous mitochondria in patient's cells (*p* < 0.05) and significantly higher number of damaged mitochondria (*p* < 0.01), in RTD *vs.* Ctrl cells. Patients' organelles are also significantly larger than those of Ctrl cells, as assessed by measuring their mean diameter.

**Figure 5:**
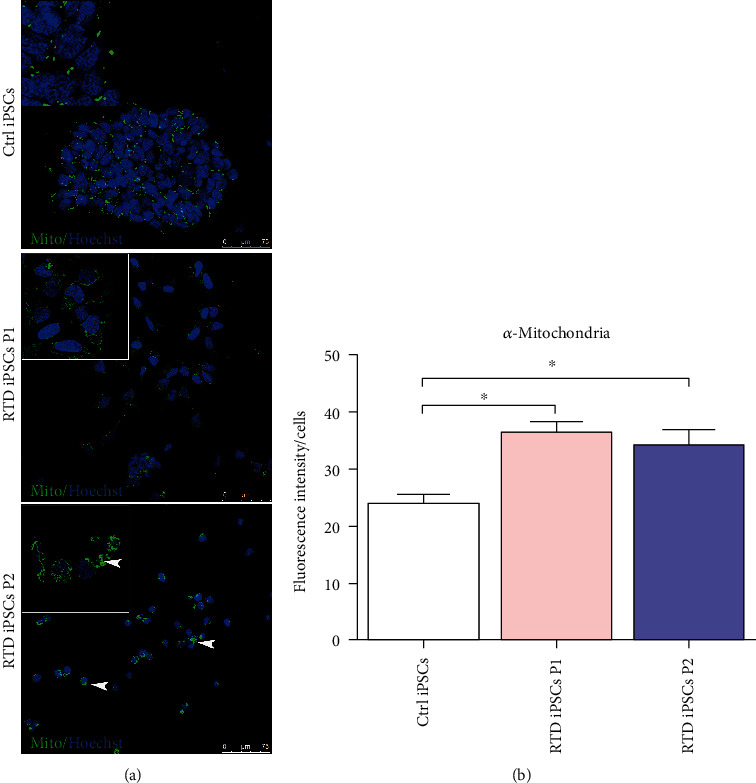
Confocal analysis of MTCO2 (Mito) in Ctrl and RTD cells: (a) immunofluorescence images of the mitochondrial marker (in green) demonstrating the higher abundance of organelles in patients' cells, as compared to controls. White arrowheads, immunoreactive aggregates; (b) fluorescence intensity levels reveal a significant increase (^∗^*p* ≤ 0.05) of *α*-mitochondria in RTD cells *vs.* Ctrl iPSCs. Nuclei are stained with Hoechst (in blue).

Aiming to further study mitochondrial distribution in our cell model, immunofluorescence with MTCO2 mitochondrial antibody was performed. Confocal analysis confirms uneven distribution and significantly greater number of these organelles in patients' cells as compared to Ctrl (Figure 5). Specifically, while P1 iPSCs show dispersed puncta, P2 cultures show immunoreactive aggregates, at a pole of the cells (arrowheads in Figure 5(a)).

#### 3.1.2. Study of Antioxidant Response of RTD iPSCs

**Figure 6:**
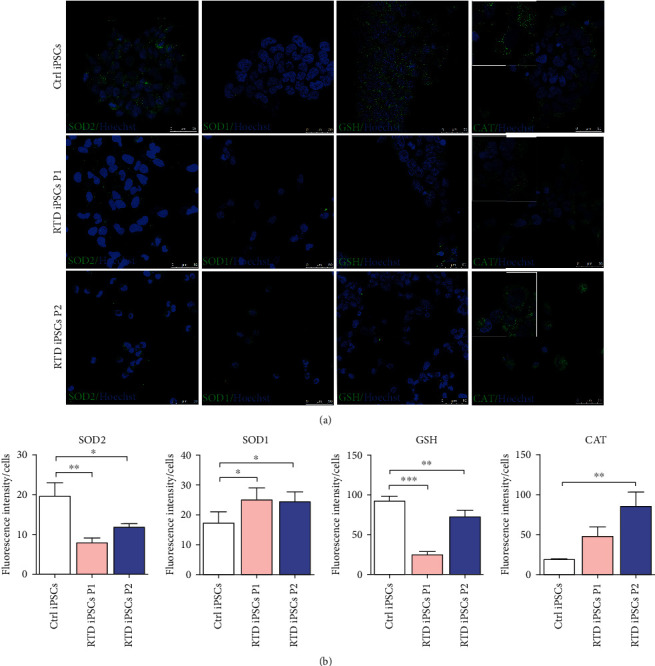
Confocal analysis of antioxidant molecules in Ctrl and RTD cells. (a) Immunofluorescence images showing finely granular SOD2 localization of higher intensity in Ctrl iPSCs with respect to patients' cells. SOD1 shows cytoplasmic immunostaining irrespective of the conditions with higher intensity in RTD cells. GSH immunolocalization appears abundantly diffused in the cytoplasmic compartment in Ctrl cells, while being sparser and occasionally aggregated in diseased iPSCs. CAT immunofluorescence is mostly granular in Ctrl iPSCs while more diffuse cytosolic staining is observed in RTD cells, especially P2 (insets). Antioxidant enzymes are marked by green fluorescence while nuclei are stained with Hoechst (in blue). (b) Fluorescence intensity analyses show statistically different expression levels of antioxidant markers. ^∗^*p* ≤ 0.05*vs*. Ctrl iPSCs; ^∗∗^*p* ≤ 0.01; ^∗∗∗^*p* ≤ 0.001*vs*. Ctrl iPSCs.

Based on ultrastructural and confocal observations, showing deranged mitochondrial features, we studied the immunofluorescence localization of Mn-dependent superoxide dismutase (MnSOD, SOD2), for its dual value as a functional mitochondrial marker and as a major ROS-scavenging enzyme. Confocal analysis reveals a significant decrease of the antioxidant enzyme in RTD iPSCs compared to Ctrl cells, which show abundant particulate SOD2 immunopositivity (Figures 6(a) and 6(b)). To gain further insights into the antioxidant response ability of RTD cells, we investigated the immunofluorescence distribution of glutathione (GSH), Cu/Zn superoxide dismutase (SOD1), and catalase (CAT). Quantification analyses reveal significantly lower expression levels of GSH in RTD than in Ctrl cells, while higher levels of SOD1 and CAT are detected (Figure 6(b)). Morphologically, SOD1 shows the typical cytoplasmic distribution, irrespective of the genotype, while GSH appears abundantly diffused in the cytoplasm of Ctrl cells and occasionally concentrated in small aggregates in RTD iPSCs. Notably, CAT localization in diseased iPSCs unexpectedly extends from peroxisome-like granules, to the cytosolic compartment.

#### 3.1.3. Study of Peroxisomal Proteins in RTD iPSCs

**Figure 7:**
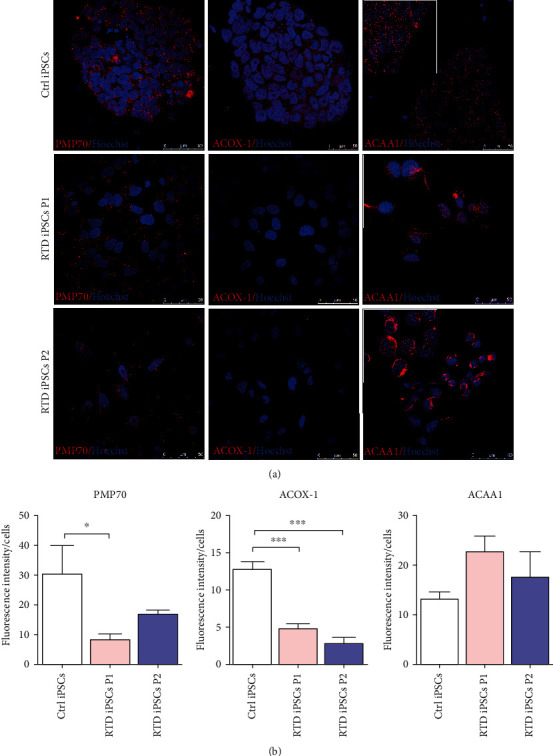
Confocal analysis of peroxisomal markers in Ctrl and RTD cells. (a) Immunofluorescence images showing finely granular immunoreactivity to PMP70 and ACOX-1, of lower intensity in RTD patients, as compared to Ctrl cells. ACAA1 distribution appears more diffuse in RTD cells (insets). Peroxisomal proteins are marked by green fluorescence, while nuclei are stained with Hoechst (in blue). (b) Fluorescence intensity analyses show statistically different expression levels of peroxisomal markers. ^∗^*p* ≤ 0.05*vs.* Ctrl iPSCs; ^∗∗^*p* ≤ 0.01*vs.* Ctrl iPSCs.

Considering RTD-associated abnormal distribution of CAT, a *bona fide* peroxisomal marker, we were prompted to extend our study to other peroxisomal markers. We chose to examine the fatty acyl translocator peroxisomal membrane protein of 70 kDa (PMP70), the first and rate-limiting enzyme of *β*-oxidation pathway Acyl-CoA Oxidase (ACOX-1), and the peroxisomal thiolase acetyl-CoA acyltransferase 1 (ACAA1), which catalyzes the last step of the cycle (Figure 7). Immunofluorescence data demonstrate downregulation of PMP70 and ACOX-1 in patients' cells, while ACAA1 shows quantitatively unchanged levels of expression. Interestingly, the immunofluorescence distribution of thiolase shows a more aggregated form, rather than the expected small granular localization (insets in Figure 7(a)).

Overall, these results are also consistent with our ultrastructural observations, suggesting the presence of fewer peroxisomes in RTD iPSCs, as compared to Ctrl cells (see Figure S1).

#### 3.1.4. Effect of RF Treatment on iPSC Morphology and Redox Status

Since empirical studies have demonstrated some amelioration of symptoms in patients treated with RF, we investigated possible beneficial effects of vitamin administration to RTD iPSCs. Based on preliminary experiments to test dose/response effect of RF treatment, we selected 1 *μ*M concentration, as the most promising dosage to be added to the medium, in terms of tolerance and detoxifying action (Suppl. Figure 2).

**Figure 8:**
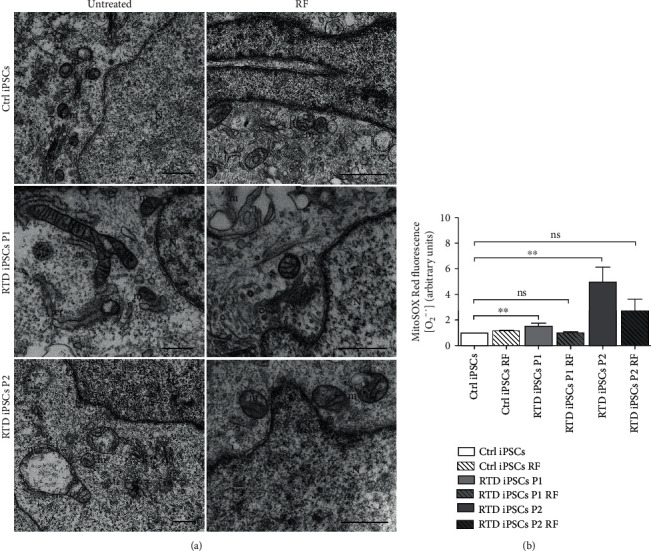
Morphofunctional effects of RF treatment on Ctrl and RTD iPSCs. (a) FIB/SEM analysis showing altered morphology in RTD vs. Ctrl iPSCs and rescue of mitochondrial ultrastructural features following RF supplementation. N: nuclei; m: mitochondria; g: Golgi complex; rer: rough endoplasmic reticulum. Scale bars, 1 *μ*m. (b) MitoSOX analysis in Ctrl and RTD cells, showing a significantly (^∗∗^*p* ≤ 0.01) higher concentration of O_2_^-.^ in patients' cells as compared to Ctrl. Redox status in RTD cells from either patient returns to Ctrl levels following RF treatment (ns: nonsignificant differences between Ctrl and RTD P1/P2+RF).

Indeed, FIB/SEM micrographs show partial rescue of morphology following such treatment, as regular mitochondrial shape and features, comparable to Ctrl cells, are observed in RF-treated RTD iPSCs (Figure 8 (a)).

For functional characterization of the diseased phenotype and possible rescue by RF, we also addressed RTD-associated redox status, by evaluating the presence of superoxide anion (O_2_^·-^), using the MitoSOX Red probe, in iPSCs, before and after RF treatment. Statistically significant changes (*p* < 0.01) in O_2_^−^ concentration in patients' iPSCs, as compared to Ctrl cells, are detected (Figure 8(b)). These alterations revert to normal levels following RF treatment.

**Figure 9:**
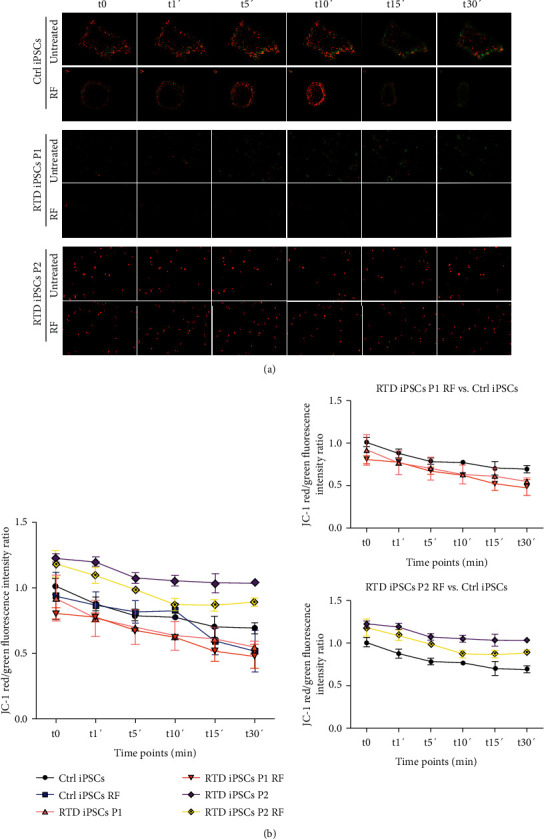
JC-1 staining images of Ctrl iPSCs and RTD iPSCs before and after RF treatment, showing the same field captured before and after 1, 5, 10, 15, and 30 min H_2_O_2_ exposure. (a) Regions of high mitochondrial polarization are revealed by red fluorescence due to J-aggregate formation, whereas depolarized regions are marked by green fluorescence of JC-1 monomers. Images show the time-dependent loss of red J-aggregate fluorescence and cytoplasmic diffusion of green monomer fluorescence following H_2_O_2_ administration. (b) Time course of cells following H_2_O_2_ exposure showing the fluorescence intensity ratio for JC-1 staining (red/green).

Furthermore, mitochondrial membrane potential (MMP) in Ctrl and RTD iPSCs was determined using JC-1 staining, under basal conditions and after H_2_O_2_ exposure. These experiments were performed before and following RF treatment. Images in Figure 9(a) show a time-dependent loss of red J-aggregate fluorescence and cytoplasmic diffusion of green monomer fluorescence, following treatment with H_2_O_2_ (after 1, 5, 10, 15, and 30 minutes). These data show significantly abnormal polarization state of RTD mitochondria, as compared to Ctrl, even though the two patients exhibit opposite trends, *i.e.*, depolarization in patient 1 and hyperpolarization in patient 2 (Figure 9). Improvement of MMP in RTD cells after RF treatment is observed, particularly in P2, which shows more efficient response to H_2_O_2_, similar to Ctrl iPSCs (Figure 9(b)).

## 4. Discussion

Riboflavin (RF) represents an indispensable nutrient for human health, as a crucial component of cellular biochemistry. Indeed, knowledge of clinical implications of its dysmetabolism is attracting growing interest. On the one hand, RF deficiency, due to either insufficient intake or disturbances in its absorption or transport, results in severe clinical conditions, while on the other, a wide array of disorders affects RF-dependent metabolic pathways. The vitamin impacts on homeostatic control of energy balance, particularly involving lipid metabolism, and redox status. RF may even exert a direct antioxidant role through its conversion of reduced to oxidized form or as a component of glutathione redox cycle [46]. RF deficiency compromises oxidant defense mechanisms by interfering with the maintenance of reduced glutathione (GSH), the master antioxidant within cells [47]. Thus, RF nutritional status impacts directly on maintaining lipid metabolism, energy metabolism, and redox balance [3]. Besides its direct metabolic role, RF acts as a regulator of gene expression at different levels, transcriptional, posttranscriptional, translational, and posttranslational [48, 49]. Interestingly, cell death and survival pathways, as well as energy metabolism, are specifically subjected to such regulation.

In the last decade, mutations in the human RF transporter genes *SLC52A2* (encoding for RFVT2) and *SLC52A3* (encoding for RFVT3) were demonstrated as causative factor for a neurodegenerative disorder referred to as riboflavin transporter deficiency (RTD), formerly known as Brown-Vialetto-Van Laere syndrome [27].

Even though several *in vivo* and *in vitro* models have been developed to dissect the molecular mechanisms underlying RF deficiency [50–52], the involvement of energy metabolism pathways occurring in specific organelles is still unclear. In the present study, we took advantage of iPSC technology, to reproduce RTD phenotype in a patient-specific cellular model.

More specifically, iPSCs derived from two patients carrying different mutations on the *SLC52A2* gene were studied from a morphofunctional viewpoint. Prior to proceeding with characterization of diseased *vs.* healthy iPSCs, we validated our model by DNA sequencing, demonstrating that RTD-associated mutations are maintained following reprogramming of fibroblasts into iPSCs. This result is in keeping with the available literature confirming dependability of iPSC technology in preserving genome integrity [32].

Overall, our data on patients' specific iPSCs demonstrate substantial defects, which may be partially reverted by RF treatment. Such abnormalities include (i) aberrant cell-cell contacts and consequent inability of cells to organize into colonies; (ii) mitochondrial dysfunction associated with ultrastructural abnormalities; (iii) altered expression of antioxidant systems; and (iv) peroxisomal alterations.

### 4.1. RTD Results in Altered Cell-Cell Contacts

As assessed by phase-contrast and electron microscopy, patients' iPSCs are unable to form regularly shaped colonies, which are instead a hallmark in iPSC cultures from healthy individuals. Lack of tight junctions and/or *zonulae adhaerentes* observed at the ultrastructural level is consistent with immunofluorescence results using the ZO-1 antibody. Indeed, confocal microscopic analysis demonstrates decreased expression and abnormal localization of this tight junction marker. Lower levels of the protein (especially in iPSCs from patient 2) may result from oxidative damage due to increased ROS production, as supported by our MitoSOX data. This interpretation would be in keeping with observations by other groups on different iPSC models of degeneration [53], showing tight junction instability and altered membrane polarity. Considering that ZO-1's primary role is to recruit other tight junction proteins (such as ZO-2 and ZO-3), forming complexes which, in turn, interact with actin filaments, disturbances in general cytoskeletal assembly and dynamics are likely to occur. On the other hand, ZO-1 localization as scattered dots or aggregates, possibly associated with lamellipodia or filopodia, rather than evenly distributed to the cell surface, suggests involvement of other cell adhesion molecules (e.g., nectin and cadherin systems), involved in filopodia extension and establishment of filopodia-filopodia contact sites [54]. Impaired cell-cell communication may also result in irregular paracellular flow with severe consequences on signaling pathways for proliferation and differentiation [55]. Relevantly, our observations well correlate with data previously obtained by RNA sequencing profiles and protein studies of the cytoskeletal constituents in RTD motor neurons, demonstrating perturbed neurofilament composition [56]. This fundamental aspect needs to be addressed in further details, to ascertain the relationship between cytoskeletal assembly and RF transport.

Noteworthy, electron micrographs demonstrate the presence of spheroidal vesicles, possibly exosomes, close to plasma membranes in RTD cells. These may be released as vectors of paracrine signals, with a protective role or as an intercellular communication mechanism, alternative to cell-cell direct contact [57].

Large vesicles are recognized next to the surface of RTD cells, especially from the most severely affected patient. These structures, reminiscent in shape and size of apoptotic bodies [58], are readily detected in SEM images and may correspond to cell blebs and fragments, containing intact organelles or nuclear portions, occasionally observed in FIB/SEM samples. Noteworthy, induction of apoptotic cell death pathway has been recently associated to RF depletion [49].

### 4.2. RTD Affects Mitochondrial Morphofunctional Features

Since flavoenzymes are responsible for a wide array of energy metabolism pathways involving mitochondria, we addressed their possible dysfunction in patient-derived iPSCs. In the present study, we demonstrate abnormally high concentration of O_2_^−^, as assessed by MitoSOX probe, in RTD cell cultures. Such redox imbalance well correlates with our ultrastructural data, showing remarkably disrupted mitochondrial morphology, consistent with the relationship linking functional and morphological state [45, 59, 60]. Indeed, taking advantage of FIB/SEM technology, we observed profoundly altered mitochondrial *crista* architecture in RTD cells. Further support to the hypothesized severely compromised energetic state in patient cells comes from JC-1 experiments, revealing abnormal mitochondrial polarization state. The key indicator of mitochondrial activity MMP is maintained at stable levels for normal cell functioning, and its prolonged perturbation, associated with changed levels of intracellular ATP, may lead to pathological consequences [61–63]. To this respect, our results on MMP, while invariably showing abnormal values consequent to RTD, intriguingly demonstrate opposite trends in the two patients: depolarization in RTD patient 1 and hyperpolarization in patient 2. Low MMP is generally associated with a failure in mitochondrial quality control, resulting from insufficient/defective systems of repair/removal of damaged organelles [64]. Whether turnover mechanisms, such as autophagy, are properly functioning in RTD condition is yet to be clarified. Ongoing work at our laboratories suggest abnormal activation of this process, in line with transcriptomic analysis performed on RTD MNs by Rizzo and coll. [56]. By contrast, mitochondrial hyperpolarization may lead to increased generation of ROS, in a self-sustaining loop detrimental to cell survival [65]. Paradoxically, hyperpolarization *per se* causes an increase of ATP demand in diseased cells [66]. While the reason for the different behavior of the two RTD cell cultures (associated with a mild or severe clinical condition) remains to be addressed, a possible explanation may refer to the different levels of CAT displayed by the two patients (see next subsection). Regardless, it should be noted that hyperpolarization and depolarization are both associated with abnormal states of respiration [64, 67]. In addition to this, we observed an increased number and size of mitochondria (as assessed by electron microscopy and *α*-mitochondria immunofluorescence analyses) in patients' derived iPSCs, and we hypothesize that this is a response of patients' cells to the altered redox status. In fact, increased number of iPSC mitochondria has also been observed in iPSCs aged in culture as a response to the aerobic environment [68].

### 4.3. ROS-Scavenging Systems Are Altered in RTD Cells

Data collected on mitochondrial morphofunctional features prompted us to address possible oxidative stress condition in RTD cells, with a focus on redox status and O_2_^−^- and H_2_O_2_-scavenging systems. This hypothesis was also driven by previous *in vivo* evidence that experimental dietary RF deficiency affects expression levels of antioxidant systems [50–52, 69–73]. We investigated the distribution of GSH and found its lower expression in RTD cells. While such decrease is easily explained by considering the implication of RF in GSH cycle, FAD is the coenzyme of glutathione reductase, which mediates regeneration of reduced GSH—it is worth noting that in diseased iPSCs, immunofluorescent dots appear sometimes aggregated. This suggests protein glutathionylation, possibly leading to proteasome/autophagic activation, an aspect that deserves further investigation.

Our confocal analysis shows significantly lower levels of SOD2 in RTD cells, as compared to their healthy counterparts. This result is in total agreement with and may in fact explain the increased number of damaged mitochondria, as assessed by FIB/SEM analysis, which are the ones presumably attacked by intraorganellar O_2_^−^ overproduction (in accordance with the MitoSOX data). We also investigated SOD1 levels, which are instead increased in RTD patients' cells, supporting the view that O_2_^−^ mostly exerts its toxic function within mitochondria. Thus, it is conceivable that cytosolic O_2_^−^ is efficiently scavenged by SOD1, however posing the additional problem, as to whether H_2_0_2_ resulting from its dismutation undergoes equally efficient removal by CAT, its major detoxifying enzyme [74, 75]. RTD cells show higher CAT levels, suggestive of a response mechanism, possibly triggered by redox sensors, such as the transcription factor NRF2. In the most severe RTD form, this induction is highly significant and may account for the unusual response to H_2_0_2_ insult, leading to the hyperpolarized state detected in JC-1 assay. To this respect, it is worth mentioning that immunofluorescence observations intriguingly suggest the presence of cytosolic CAT, besides its expected peroxisomal localization. Such uncommon distribution may in fact reflect the susceptibility to oxidative stress of CAT import machinery, involving a noncanonical PTS1 signal. Specifically, the peroxisomal import receptor peroxin 5 (PEX5) was shown to function as a stress sensor, retaining CAT in the cytosol in oxidative stress conditions [76]. Interestingly, the same authors bring evidence that cytosolic CAT can protect against H_2_O_2_-mediated redox changes, thus constituting a cellular defense mechanism to combat oxidative insults of extraperoxisomal (possibly including mitochondrial) origin.

### 4.4. Downregulation of Peroxisomal Proteins in RTD Cells Suggests Lipid Dysmetabolism

Interplay of mitochondria with peroxisomes is receiving growing attention, in view of their cooperative roles in regulating redox signaling pathways and in sustaining metabolic homeostasis. In addition to ROS metabolism, to which both scavengers (namely, CAT, classical peroxisomal marker) and ROS-producing oxidases contribute, peroxisomes are responsible for other energy metabolism pathways, such as fatty acid *β*-oxidation. We addressed the involvement of acyl-CoA oxidase (ACOX-1) and 3-oxoacyl-CoA thiolase (ACAA-1, thiolase), catalyzing the first and the last step of peroxisomal *β*-oxidation, respectively. Based on confocal analysis, downregulation of ACOX1 in patients' cells, as compared to Ctrl, is demonstrated, while thiolase levels are unchanged. Since ACOX-1 is the rate-limiting enzyme of peroxisomal *β*-oxidation cycle, overall decreased efficiency in metabolizing very long-chain fatty acids likely occurs. Such an impairment would also result from limited availability of the obligate cofactor FAD, due to RTD condition. Even data collected for PMP70, a major component of peroxisomal membranes and crucial fatty acid translocator, argue for lipid dysmetabolism. In fact, confocal microscopic analysis reveals a decreased immunoreactivity, possibly indicating the lack of import-competent peroxisomes. Moreover, the ultrastructural analysis aimed at identifying the peroxisomal population supports a relatively low presence of peroxisomes in RTD cells. Besides, disturbed peroxisomal biogenesis would also be supported by mislocalization in RTD cells of CAT (cytosolic) and ACAA1 (as large aggregates), possibly driven by oxidative damage and cytoskeletal derangement. To this respect, it is worth mentioning that our knowledge of the peroxisome interaction with the cytoskeleton has dramatically increased. Indeed, attachment of peroxisomes to cytoskeleton and movement along microtubular filaments and actin cables are essential and highly regulated processes enabling metabolic efficiency, biogenesis, maintenance, and inheritance of this dynamic cellular compartment [77].

The observed downregulation of peroxisomal lipid metabolism in RTD cells well correlates with *in vivo* experimental studies demonstrating the negative effect of RF deficiency on *β*-oxidation enzyme activity [71, 72, 78]. On the other hand, our data are consistent with the proposed role of RF in controlling lipid metabolism pathways through gene expression regulation at diverse levels [43, 44].

### 4.5. RF Treatment Restores RTD Cell Phenotype and Redox Status

Clinical studies by Bosch and coll. [23] brought evidence that therapeutic use of RF extends RTD patients' life expectancy, ameliorating neurological symptoms. Based on these empirical studies and on our results on redox imbalance, we performed RF treatment on patient-specific iPSCs. Vitamin supplementation ameliorates RTD phenotype, particularly as regards mitochondrial ultrastructure. Consistently, such treatment was able to revert O_2_^−^ concentration, as assessed by MitoSOX assay, to normal levels. RF ability in restoring redox status is compatible with its proposed ROS-scavenging role, through an either direct or indirect action, as a component of glutathione redox cycle [46, 47, 79, 80].

Such beneficial effects support the hypothesized cooperation among different RFVT isoforms, particularly involving, in our iPSC model, dimerization of RFVT1 and RFVT2 to accomplish RF uptake [44]. Nonetheless, we cannot rule out some residual uptake activity of the mutated protein. Further studies are needed to ascertain these hypotheses.

## 5. Conclusions

Human iPSCs represent an innovative model for numerous clinical studies, opening new perspectives of research and applications in the biomedical field. Moreover, the generation of iPSCs from patients affected with rare genetic disease allows to overcome ethical and technical problems due to manipulation of stem cells, allowing for site-specific genetic correction. In the present study, we took advantage of iPSC technology to shed light on the morphofunctional mechanisms underlying RTD syndrome, a rare neurodegenerative disorder caused by loss-of-function mutation in two RF transporter genes.

**Figure 10:**
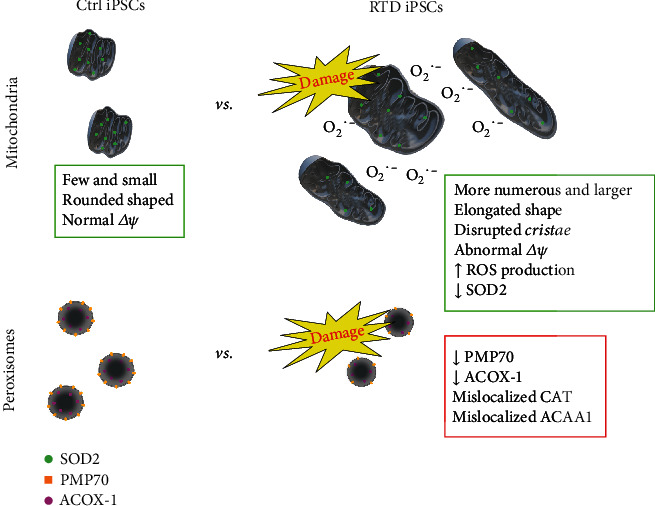
Conceptual schema showing morpho-functional changes related to mitochondria and peroxisomes observed in RTD iPSCs.

While supporting the innovative potential of our iPSC model of RTD, our work demonstrates concurrent mitochondrial/peroxisomal alterations in the disease. The dramatic changes here reported range from cell junctional contacts, possibly involving cytoskeletal assembly, to altered MMP, with consequent oxidative stress and antioxidant response, to disturbed peroxisomal biogenesis and metabolism (Figure 10). Since our study represents to our knowledge the first to address the distribution of peroxisomal population in iPSCs, further investigation on this topic is encouraged, especially in view of the interplay with the cytoskeleton and other cell compartments, namely, ER and mitochondria [77, 81, 82].

Our findings, highlighting multiple defects contributing redox imbalance and energy dysmetabolism in RTD syndrome, may also explain specific manifestations of different diseases sharing defects in RF absorption or metabolism. Also, extending to the cellular level, the empirical studies by Bosch and coll. [23] will hopefully open new perspectives in dissecting the pathomechanisms of disease and in refining patient-specific, RF-based therapeutic strategies.

## Data Availability

The data used to support the findings of this study are either included within the article or available from the corresponding author upon request.

## Supplementary Materials

Supplementary materials Figure S1: ultrastructural features of iPSCs from Ctrl and RTD patients, with reference to the presence of peroxisomes. Figure S2: dose-response effect of RF treatment assessed by MitoSOX Red assay.
