# Supplementary material for: Mitochondrial and Peroxisomal Alterations Contribute to Energy Dysmetabolism in Riboflavin Transporter Deficiency
Source: Oxid Med Cell Longev. 2020 Aug 12;2020:6821247. doi: 10.1155/2020/6821247 (PMC7443020; doi:10.1155/2020/6821247)
Supplement: Supplementary materials — Figure S1: ultrastructural features of iPSCs from Ctrl and RTD patients, with reference to the presence of peroxisomes. Figure S2: dose-response effect of RF treatment assessed by MitoSOX Red assay. [file 6821247.f1.pdf]

Supplementary Figure 1

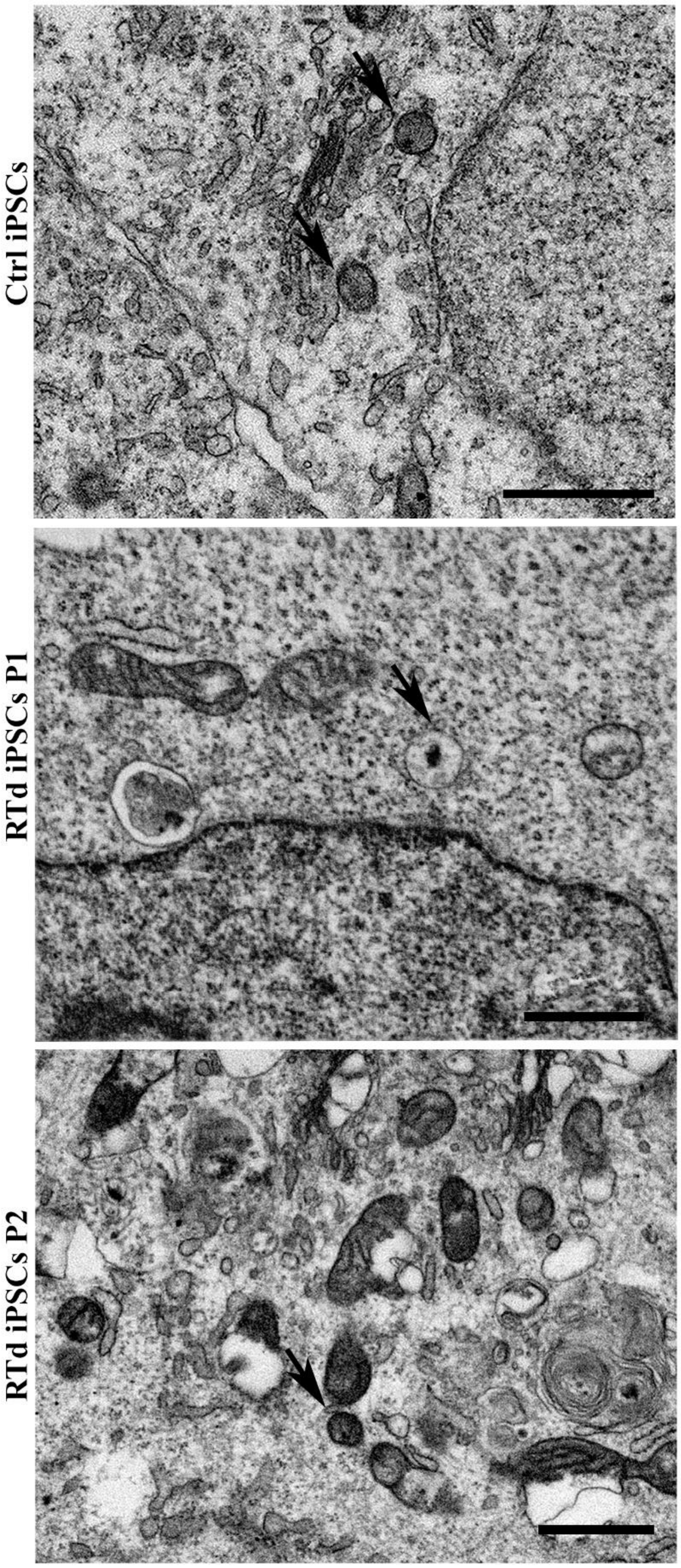

Figure S1: FIB/SEM micrographs showing peroxisomes (Black arrows) in Ctrl iPSCs and RTD cells. These organelles are hardly detectable in RTD cells.

Supplementary Figure 2

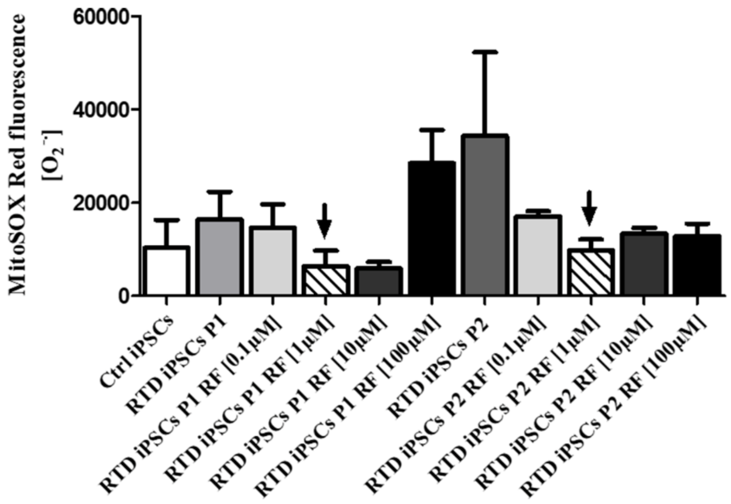

Figure S2: Dose-response effect of RF treatment assessed by MitoSOX Red assay. Black arrows indicate riboflavin 1 µM, which can lower mitochondria superoxide anion levels.
